# Mold-Face Heating Mechanism, Overflow-Well Design, and Their Effect on Surface Weldline and Tensile Strength of Long-Glass-Fiber-Reinforced Polypropylene Injection Molding

**DOI:** 10.3390/polym12112474

**Published:** 2020-10-25

**Authors:** Po-Wei Huang, Hsin-Shu Peng, Wei-Huang Choong

**Affiliations:** 1 Ph.D. Program of Mechanical and Aeronautical Engineering, Feng Chia University College of Engineering and Science, Taichung 40724, Taiwan; bowei8915@gmail.com (P.-W.H.); colechoong.mcaefcu@gmail.com (W.-H.C.); 2 Mechanical and Computer Aided Engineering, Feng Chia University College of Engineering and Science, Taichung 40724, Taiwan

**Keywords:** long-glass-fiber-reinforced polypropylene, mold-face heating, surface roughness, tensile strength, weldline

## Abstract

Long-fiber polymers offer the advantage of a lower production cost because specific tool designs are required for conventional injection molding equipment to produce long-fiber polymer parts. The use of long fibers allows relatively high fiber aspect ratios to be obtained, thereby enhancing composite stiffness, strength, creep endurance, and fatigue endurance. However, the multigate design of the injection-molded part can result in weldline formation during the molding process, which reduces the structural strength of the molded part. Therefore, in this study, the surface quality, fiber compatibility, and structural strength of long-glass-fiber-reinforced polypropylene (PP/LGF) injection-molded samples were compared in the use versus nonuse of a mold-cavity overflow-well area and the mold-face infrared heating method. The experimental results indicate that the mold-cavity overflow-well area more greatly improved the surface roughness of the PP/LGF molded samples. Moreover, the infrared heating of the mold-face decreased the weldline depth of the samples. Optical-microscopy images and mold-cavity pressure distributions indicated that the weldline tensile strength and the interface compatibility between fibers and melts at the weldline region during the molding stage were higher in the use than in the nonuse of the mold-cavity overflow-well and mold-face infrared heating method.

## 1. Introduction

In recent years, fiber-reinforced polymers, which have excellent mechanical properties, have become the preferred composite material for executing lightweight applications in many industries, such as the automotive industry. Due to their advantages such as high strength or stiffness, design flexibility, and ability to be recycled, the aforementioned polymers have been widely used to construct structural parts [1,2,3,4,5,6,7]. The fiber length, content, and orientation of the polymers play crucial roles in mechanical property enhancement [2,8,9]. Long-fiber-reinforced polymer (Lf-P) composites have attracted considerable research attention due to their desirable features, such as high strength, stiffness, and impact properties, which are superior to those of short-fiber-reinforced polymer composites [10,11,12]. Lf-P composites consist of a polymer matrix and reinforcing fillers with an aspect ratio of approximately 100–3000 [13,14]. Injection molding is one of the most favorable polymer processing methods because polymeric products can be manufactured rapidly at relatively low cost [11,15,16]. However, the fibers incorporated into fiber-reinforced composites are damaged by shear forces at high temperatures during the injection molding process. Furthermore, such damage can accumulate, thereby significantly reducing fiber length. As fiber length increases, the possibility of the composite being cut also increases. Consequently, the glass-fiber length of the final fiber-reinforced product is different from that of the original pellet length. Fiber breakage is mainly influenced by the fiber’s characteristics, as well as by the processing technology and forming equipment used [2,5,9,17,18,19,20,21]. Studies have suggested that most fiber breakage occurs in the screws and nozzles during injection molding process. Similarly, in the process of mold filling, some degree of fiber breakage occurs because of the strong shear forces acting on the fibers [8,22]. The physical performance of injection-molded composites is determined by the final, not original, fiber length. Moreover, the injection molding process involving high shear intensity can affect the thermal conductivity of the composites, likely also influencing the fiber orientation. The process conditions and geometry of the mold used to manufacture the composite can affect the microstructure (length, orientation, and dispersion of the fibers) of the Lf-P composite. Simultaneously, the mechanical properties are influenced by the microstructure of the Lf-P composite [9,23,24,25]. Artifacts molded from fiber-reinforced composites may have a visually undesirable aspect of fibers being present on the product surface. The surfaces of products obtained from fiber-reinforced composites tend to exhibit white spots caused by the exposed fibers. The formation of white spots may limit the application of fiber-reinforced composites when visual appeal is required. The formation of white spots can be controlled if suitable process parameters are used during the processing of the composite. Moreover, high performance levels can only be obtained when the composite part contains as a high fiber concentration and when the reinforcing fibers in the final product have a sufficiently high aspect ratio (length/diameter). However, the demands of mass production are often in conflict with the retention of high aspect ratios and the use of high fiber concentrations [8,9,10,13,26,27,28]. Long fibers provide a final product with superior mechanical properties and surface quality [20,29]. During injection molding of long-fiber-reinforced thermoplastic, fiber interactions are complicated, with long fibers sometimes behaving as short fibers due to reinforcement tangling, which may negatively affect fiber orientation. Even if a multilayered structure is maintained, the core-layer thickness increases with the fiber length, which suggests a different mechanism [14,30].

This study explored the effects of the compatibility between melts and fibers through a double-gate design on the surface quality of molded samples. Moreover, a mold with a mold-cavity overflow-well and a mold-face infrared heater was designed to investigate the surface roughness, weldlines, and tensile properties of molded long-glass-fiber-reinforced polypropylene (PP/LGF) composite samples obtained through the injection molding process.

## 2. Materials and Methods

### 2.1. Materials

The PP/LGF composite was manufactured by GRECO (Great Eastern Resins Industrial Co. Ltd.; Taichung, Xitun District, Taiwan; material number LGP50-12). Glass fibers with a diameter of 17 μm were surface-treated with a coupling agent. The average length and content of the reinforcement fibers in the received composite pellets were 12 mm and 30 wt.%, respectively. Before processing, the PP/LGF composites were heat-preserved, dehumidified, and dried at 50 ± 5 °C in a dehumidifier with forced heat circulation.

### 2.2. Mold and Test Samples

**Figure 1:**
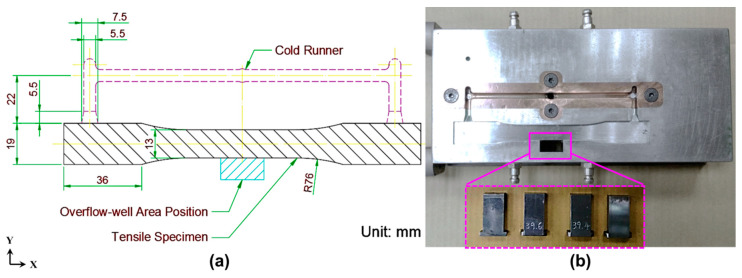
Schematic of tensile specimens: (**a**) diagram of the mold cavity of specimens; and (**b**) configuration of overflow-well area and insert.

A double-gate design tensile specimen mold (Figure 1a) with a thickness of 3.6 mm was designed for the experiment, allowing molded specimens with weldlines to be acquired. Conventionally stipulated specimen dimensions can vary considerably depending on the experimental requirements, as described in the Annual Book of ASTM Standards. In this study, tensile specimens with the ASTM-D638 standard were used to determine the surface quality, fiber compatibility, and tensile characteristics of PP/LGF. Furthermore, an overflow-well was used to observe the weldline interface between melt and fiber. During the filling stage, the frozen layers had a higher percentage due to the lower mold-face temperature; thus, by limiting the bonding rate at the weldline interface, an overflow-well area (20 mm × 10 mm) was designed with exchangeable overflow-well depths of 0.9, 1.8, and 3.6 mm (Figure 1b).

### 2.3. Infrared Heater for Heating Mold-Face

**Figure 2:**
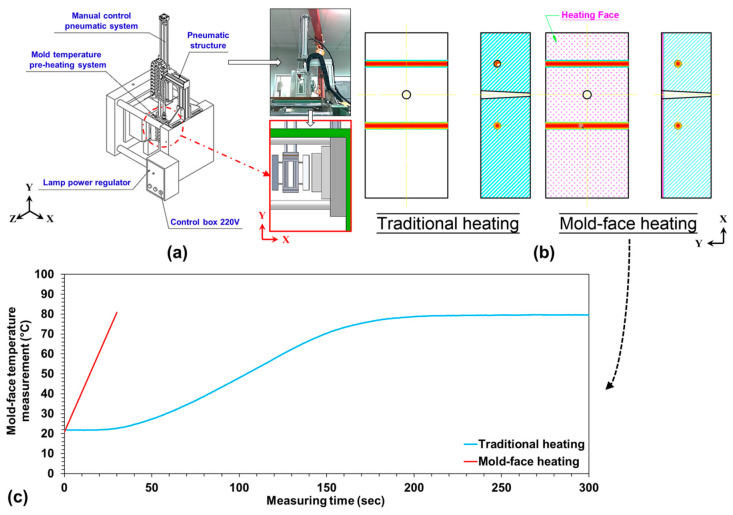
Design and application of infrared heater: (**a**) schematic of the infrared heater; (**b**) heating mechanisms when using traditional and infrared heating methods; and (**c**) trend of mold-face temperature when using traditional and infrared heating.

Injection-molded parts are currently required to have high surface quality. In the molding process, the generation of surface defects, such as weldlines or flow marks, should be appropriately controlled because these defects can worsen the molded part’s appearance. In this study, attempts were made to decrease the surface roughness and increase the weldline tensile strength of injection-molded samples. An infrared heater (Figure 2a) was designed and used to heat the mold-face to achieve a high mold-face temperature. The heating element included two fast medium-wave infrared light fixtures (temperature range of 1400–1800 °C and wavelength of 1.4 μm). The power of each infrared heating lamp was 1200 W (resulting in a total heating power of 2400 W). To observe whether an appropriate mold-face temperature was reached, an infrared thermal imager was used. Many molding problems and surface defects can be eliminated when using a high mold temperature. The cheapest method of achieving a high mold temperature is to run cooling water close to 100 °C. When a mold temperature higher than 100 °C is required, a high-pressure water supply system (which prevents steaming) or heating oil can be used. Such systems may introduce channel connection damage, thereby compromising safety after long-term operation. When the water temperature is set to 100 °C on the mold temperature controller, the actual temperature of the mold-face only reaches 80 ± 5 °C. Moreover, the heating time of the mold-face and the cooling time of the product are relatively long. The low heat transfer coefficient of heating oil may result in low energy efficiency. Local mold heating with electric heating elements is sometimes used to facilitate mold temperature control. However, this method incurs additional design and tool costs. The rapid heating system constructed in this study using an infrared device can achieve a lower heating duration, energy consumption, and cost than heating methods using hot water, heating oil, or electric cartridge heaters. Figure 2b presents a comparison of the temperature distribution and time for heating the mold-face when using traditional water heating and infrared heating (Figure 2c). The temperature difference observed at the mold-face after heating is lower when performing rapid heating with an infrared heater than when using the aforementioned heating methods [31].

### 2.4. Test Methodology

**Figure 3:**
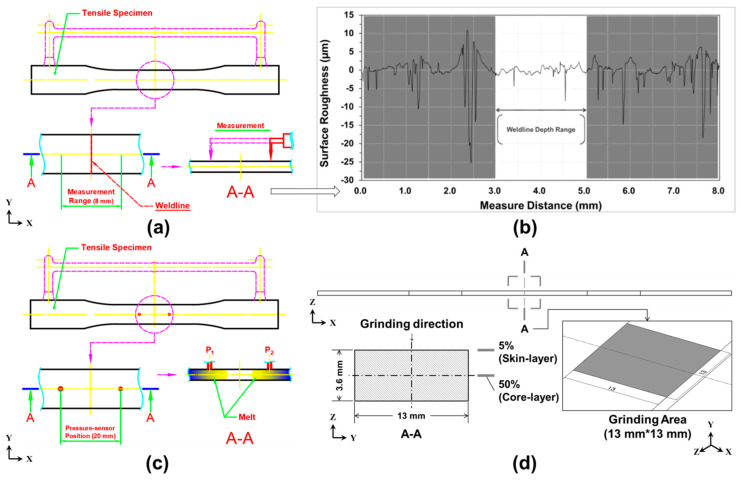
Schematics of surface measurement and pressure sensor position: (**a**) schematic of surface roughness measurement; (**b**) schematic of weldline depth measurement; (**c**) schematic and position of mold-cavity pressure sensor; and (**d**) schematic and position of grinding stage.

In this study, a double-gate design and a mold-cavity overflow-well area were used to test the weldline molding of the molded samples and observe the surface quality of samples with weldlines. Moreover, a mold-face infrared heater was designed to observe the change in form and strength of the weldline, whereby the infrared heater was used to heat the mold-face thereby allowing an examination of the effect of fiber-melt compatibility. The mold-face infrared heater was lowered into the mold, and the mold-face temperature was set to 100 °C. The mold-face was heated from its initial value to approximately 100 °C (heating duration 30 s). The observation of surface quality was divided into two parts. First, an instrument for measuring surface roughness was used (in Ra; SJ-310, Mitutoyo, Taichung, Taiwan). The macroscopic surface roughness was measured by offsetting the specimens by 4 mm to the right and left of their center positions (total measurement distance 8 mm; Figure 3a). Second, the weldline position was examined microscopically, and the weldline depth was measured when two melts were sewn (Figure 3b). In addition, mold-cavity pressure sensors were embedded at the site where both melts were merged. The measurement points were offset by 10 mm above and below the centerline of the tensile specimen to investigate the pressure trend of the two melts at the specimen flow end (Figure 3c). The correlation between the flow-end pressure variation and weldline depth was determined using the overflow-well and mold-face heating. To examine the compatibility between the fibers and melts, the specimens were ground and observed through optical microscopy. The specimens were ground surface-forward and offset by 5 mm to the right and left of their center position (total measurement distance 10 mm; grinding along the *Z*-direction of the test specimen, i.e., from the skin layer to the specimen’s core layer with grinding thickness and positions at 5% and 50%; Figure 3d). Tensile strength test was determined on an AG-100KN machine with a full load of 1000 kg/f at a displacement rate of 20 mm/min. In total, 20 samples molded under the same molding conditions were used for each type of testing. The mean value of the 20 samples was used for analysis. The sample dimensions varied considerably depending on the experimental requirements. The tensile test was conducted according to the ASTM-D638 standard. The samples broke around their center portion despite the existence of a weldline. Table 1 presents the overflow-well depth and mold-face temperature in each experimental condition.

## 3. Results and Discussion

### 3.1. Surface-Floating Fibers and Roughness of the Samples

**Figure 4:**
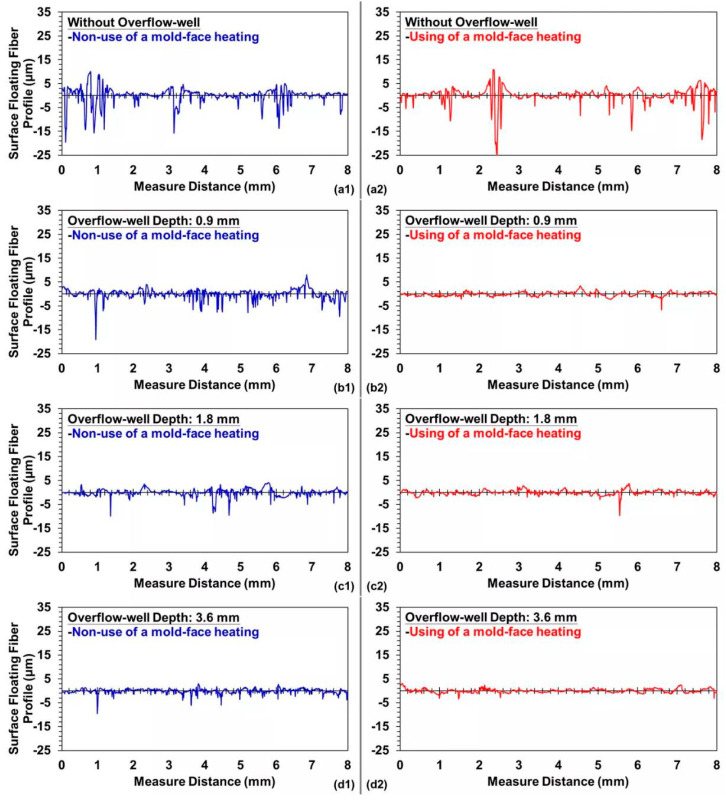
Variation in the presence of surface-floating fibers with and without the use of mold-face heating: (**a1**) and (**a2**) without overflow-well; (**b1**) and (**b2**) with an overflow well having a depth of 0.9 mm; (**c1**) and (**c2**) with an overflow-well having a depth of depth of 1.8; and (**d1**) and (**d2**) with an overflow-well having a depth of 3.6 mm.

**Figure 5:**
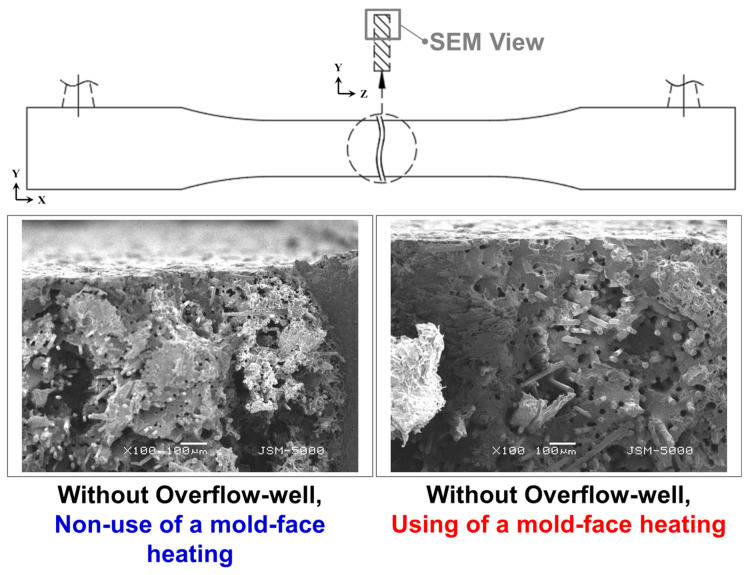
Scanning Electron Microscope (SEM) images of samples with and without the use of mold-face heating.

**Figure 6:**
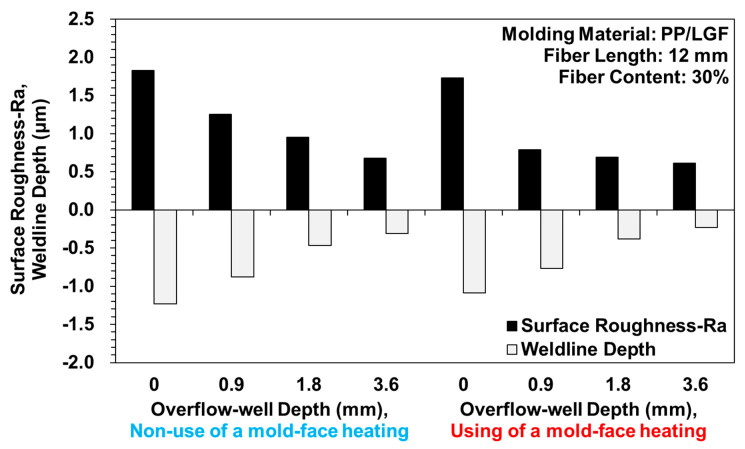
Measured trends of surface roughness (Ra) as a function of weldline depth.

Samples with weldlines were molded using different overflow-well depths and types of mold-face heating, whereby the surface roughness or the presence of surface-floating fibers was observed after two melts were merged (Figure 4). In the molding process conducted without using an overflow-well, poor compatibility was observed between the fibers and melts. Moreover, a strong surface-floating fiber phenomenon was noticed, which weakened as the overflow-well depth increased (Figure 4b1, b2, c1, c2, d1, d2) due to overflow-well provided an important influence on melt flow during filling stage and provided a better molecular chain entanglement for molded samples. Moreover, after the use of mold-face heating, the melt flow resistance decreased with an increase in the mold-face temperature. The surface-floating fiber phenomenon weakened when the compatibility between melts increased (Figure 5). Figure 5 displays the melt containing fiber during the filling process with the heating mold-face, improved the surface-floating fiber on the skin-layer of the molded samples. Due to the high shear region around the surface of the molded sample during filling process, the shear heating phenomenon will cause partial differences in the fluidity of the melt and the melt close to the mold-face has poor fluidity. Under normal mold temperature conditions, as the mold-face temperature is low, the melt solidifies instantly on the skin-layer, allowing the fibers to insert into the skin-layer at a certain angle. Increasing mold temperature is an effective way of improving surface appearance. Mold heater was used by Wang [32,33] to improve the floating fibers and eliminate the weldlines. Therefore, after heating the mold-face, the melt solidification phenomenon and fiber alignment can be improved (Figure 6). Figure 6 displays the trends in measured surface roughness (Ra) and weldline depth. The cavity volume increased with overflow-well depth. The melt flow length increased with mold-cavity volume, which in turn increased with the compatibility between melts. To enhance the compatibility between the fibers and melts, the presence of surface-floating fibers was improved and the surface roughness was reduced. The results obtained with and without an overflow-well were compared. When using an overflow-well with a depth of 0.9 mm, the surface roughness decreased by 31.93% compared with that obtained without an overflow-well. In addition, after mold-face heating was performed, the melt flow resistance decreased with mold-face temperature. The quantity of surface-floating fibers decreased to 5.65% when compatibility was achieved between two melts after mold-face heating (without an overflow-well). Dents were observed during the compatibility process of the two melts after surface roughness measurement. The surface roughness data are selectively enlarged in Figure 4. The weldline depth decreased with an increase in the overflow-well depth. Thus, when using an overflow-well, the compatible portion of the two melts was transferred to the overflow-well, and the size of the weldline dent in the samples decreased. When using an overflow-well with a depth of 0.9 mm, the weldline depth decreased by 38.48% compared with that obtained without an overflow-well. In addition, after mold-face heating, the melt flow resistance and the size of the weldline dent decreased with an increase in the mold-face temperature. After mold-face heating, the weldline depth decreased to 13.45% (without an overflow-well).

### 3.2. Strength Variation and Fiber Compatibility Assessment

**Figure 7:**
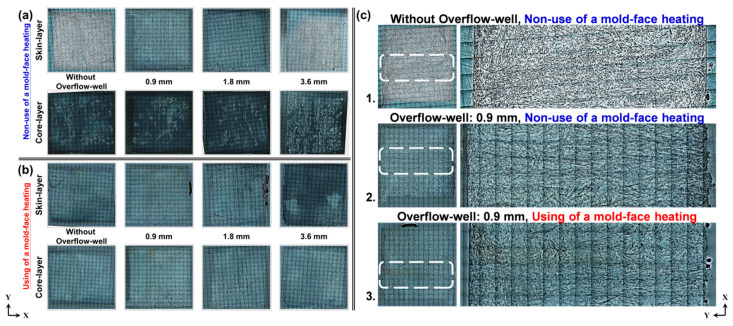
Optical-microscopy (OM) images of samples with different overflow-well depth and mold-face temperatures: (**a**) level of incompatibility between fibers and melts as a function of overflow-well depth and fiber orientation; (**b**) level of incompatibility between fibers and melts as a function of use-mold-face heating and fiber orientation; and skin layers of samples: (**c1**) without overflow-well and mold-face heating; (**c2**) using an overflow-well with a depth of 0.9 mm without mold-face heating; (**c3**) using an overflow-well with a depth of 0.9 mm and mold-face heating.

Figure 7 presents optical-microscopy (OM) images of the fiber orientation after compatibility was achieved between the two melts. By using an overflow-well and mold-face heating in the molding process, the flow direction of the melt and the fiber orientation could be changed. Figure 7a displays a comparison of the molding process with and without an overflow-well. The fibers were turbulent and exhibited entanglement and uneven dispersion when an overflow-well was not used. By contrast, when using an overflow-well, the phenomenon of fiber turbulence was weakened, while the dispersion of fibers and their compatibility with the melt improved. In addition, for the weldline tensile samples obtained through mold-face heating, the flow resistance was small when the melt was filled due to the uniform surface temperature of the mold, and the dispersion of fibers and their compatibility with the melt improved (Figure 7b). Figure 7c displays skin layers of samples with different mold-face temperatures when using and not using an overflow-well with a depth of 0.9 mm. The weldline phenomenon was obviously improved with two-melt compatibility (Figure 7c1, c2). Due to the high mold-face temperature and uniform temperature distribution, the flow resistance of the melt was low during the filling process; the fibers did not solidify easily in the skin layer, in addition to having an uneven temperature distribution; and the presence of surface-floating fibers was lessened (Figure 7c3). Moreover, the compatibility of the fibers with the melts increased when the overflow-well was used.

**Figure 8:**
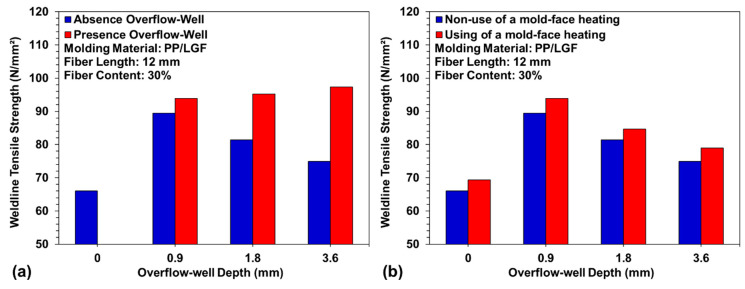
Variation of weldline tensile strength under different overflow-well depth: (**a**) absence and presence of overflow-well; and (**b**) non-use and use of mold-face heating process.

**Figure 9:**
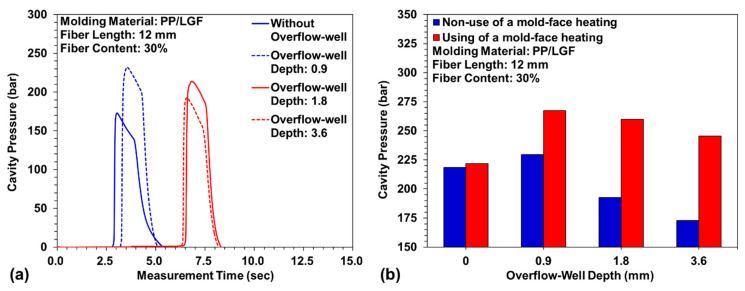
Variation of cavity pressure: (**a**) absence and presence of overflow-well; and (**b**) non-use and use of mold-face heating process.

PP/LGF composites were processed with an overflow-well of different depths to examine the weldline tensile strength on the sample. Figure 8a displays the measurement data for tensile strength of the weldline with the use and nonuse of the overflow-well area. As the overflow-well depth increased, the weldline tensile strength increased. When the overflow-well area was removed, the weldline tensile strength decreased. The weldline tensile strength obtained with the use of the 0.9 mm deep overflow-well area was 43.54% higher than that obtained with the nonuse of overflow-well area. Moreover, the weldline tensile strength obtained with the 0.9 mm deep overflow-well increased by 4.76% when the mold-face was subjected to high-temperature heating. The weldline tensile strength obtained when the 1.8 mm deep overflow-well area was used without mold-face heating was 16.2% lower than that obtained with mold-face heating. The weldline tensile strength obtained when the 3.6 mm deep overflow-well area was used without mold-face heating was 23.5% lower than that obtained with mold-face heating. In the weldline formation process between melts, their compatibility with fibers and the weldline tensile strength increased with an increase in the mold-cavity volume (caused by an increase in the depth of the overflow-well area). However, when the overflow-well area was removed, the tensile area was reduced, and this increased the tightness between fibers where the overflow-well area was located. The weldline tensile strength decreased with an increase in the depth of the overflow-well area. An injection molding experiment was also conducted with a mold-face heater. Subsequently, the changes in weldline tensile strength with the use and nonuse of mold-face heating were compared, as well as the changes caused due to the presence and absence of the overflow-well. In addition, during the weldline formation of two melt strands, and with the combination of the mold-face heater and the changes in mold-face temperature with overflow-well depth, the effects of the use and nonuse of mold-face heating were observed. Figure 8b indicates that mold-face heating significantly affects the weldline tensile strength. The weldline tensile strength decreased as the depth of the overflow-well area increased. The experimental results indicate the successful formation of a weldline between melts, whereby a thin overflow-well area and high mold-face temperature increased the flow ability of the melt and changed the cross-sectional area. The fibers packed in the overflow-well area improved the compatibility between fibers and melts, which increased the weldline tensile strength. Therefore, in this study, a cavity pressure sensor was embedded at the position where the two melts merged. Two points above and below the centerline were used as measurement points (Figure 3c). The overflow-well area significantly affected the weldline tensile strength. Figure 9a displays the measurement data related to cavity pressure for the use versus nonuse of an overflow-well area. As the depth of the overflow-well area increased, the pressure at the flow end decreased. An increase in the mold-cavity volume led to a decrease in pressure at the flow end. When the mold-face was heated to a high temperature, the fluidity of the melt increased. When the depth of the overflow-well area increased, the flow-end pressure tended to increase (Figure 9b).

## 4. Conclusions

This study used the mold-face infrared heating method to examine the presence of surface-floating fibers (roughness, Ra) and determine the weldline depth of specimens. Moreover, an overflow-well was used to study the compatibility of two melts with the fibers. The findings of this study are as follows:

1. The surface-floating fibers of the PP/LGF samples fabricated using a double-gate molding design varied with the overflow-well depth. the molding process conducted without using an overflow-well, poor compatibility was observed between the fibers and melts, and, after the use of mold-face heating, the melt flow resistance decreased with an increase in the mold-face temperature. The surface-floating fiber phenomenon weakened when the compatibility between melts increased.
2. Samples with weldlines were molded using different overflow-well depths and types of mold-face heating. The cavity volume increased with overflow-well depth. The melt flow length increased with mold-cavity volume, which in turn increased with the compatibility between melts. The surface roughness obtained when using an overflow-well with a depth of 0.9 mm was 31.93% lower than that obtained when not using the overflow-well.
3. When using an overflow-well during the high-temperature heating of the mold-face, the melt was not hindered in the filling process, the fibers flowed relatively easily, the phenomenon of fiber turbulence was weakened, and the dispersion of fibers and their compatibility with the melt improved. For the weldline tensile samples obtained through mold-face heating, the flow resistance was small when the melt was filled due to the uniform surface temperature of the mold.
4. The data for the tensile strength of the weldline with the use and nonuse of the overflow-well area were measured. The weldline tensile strength increased with the increase of overflow-well depth. When the overflow-well area was removed, the weldline tensile strength decreased and the weldline tensile strength obtained with the 0.9 mm deep overflow-well was 43.54% higher than that obtained without the overflow-well. Moreover, the weldline tensile strength obtained with the 0.9 mm deep overflow-well increased by 4.76% when the mold-face was subjected to high-temperature heating.

## Figures and Tables

**Table 1:** Configuration of the overflow-well and mold-face heating for the molding of long-glass-fiber-reinforced polypropylene (PP/LGF) samples.

Parameters							
Controllable Factors					Factors Maintained Constant		
Use versus nonuse of mold-face heating	Nonuse		Use		Injection pressure (bar)		70
					Packing pressure (bar)		20
					Cooling time (s)		20
Overflow-well depth (mm)	0	0.9	1.8	3.6	Mold heating, water temperature (°C)		80
**Random Order of the Treatments for Molding the Samples**							
**Method**	**Overflow-Well Depth**					**Mold-Face Temperature**	
1	0					Nonuse	
2	0.9					Nonuse	
3	1.8					Nonuse	
4	3.6					Nonuse	
5	0					Use	
6	0.9					Use	
7	1.8					Use	
8	3.6					Use	

## References

[1] Duan S., Zhang Z., Wei K., Wang F., Han X. (2020). Theoretical study and physical tests of circular hole-edge stress concentration in long glass fiber reinforced polypropylene composite. J. Compos. Struct..

[2] Hou X.Q., Chen X.Y., Liu B.C., Chen S.C., Li H.M., Cao W. (2019). Fracture and orientation of long-glass-fiber-reinforced polypropylene during injection molding. J. Polym. Eng. Sci..

[3] Zhang Q., Zhang J., Wu L. (2018). Impact and energy absorption of long fiber-reinforced thermoplastic based on two-phase modeling and experiments. Int. J. Impact Eng..

[4] Giusti R., Zanini F., Lucchetta G. (2018). Automatic glass fiber length measurement for discontinuous fiber-reinforced composites. J. Compos. Part A Appl. Sci. Manuf..

[5] Azenha J., Gomes M., Silva P., Pontes A.J. (2018). High strength injection molded thermoplastic composites. J. Polym. Eng. Sci..

[6] Duan S., Mo F., Yang X., Tao Y., Wu D., Peng Y. (2016). Experimental and numerical investigations of strain rate effects on mechanical properties of LGFRP composite. J. Compos. B Eng..

[7] Duan S., Tao Y., Han X., Yang X., Hou S., Hu Z. (2014). Investigation on structure optimization of crashworthiness of fiber reinforced polymers materials. J. Compos. B Eng..

[8] Kallel T.K., Taktak R., Guermazi N., Mnif N. (2018). Mechanical and structural properties of glass fiber-reinforced polypropylene (PPGF) composites. J. Polym. Compos..

[9] Thomason J.L. (2008). The influence of fibre length, diameter and concentration on the modulus of glass fibre reinforced polyamide 6,6. J. Compos. Part A Appl. Sci. Manuf..

[10] Lee T.W., Lee S., Park S.M., Lee D. (2019). Mechanical, thermomechanical, and local anisotropy analyses of long basalt fiber reinforced polyamide 6 composites. J. Compos. Struct..

[11] Seong D.G., Kang C., Pak S.Y., Kim C.H., Song Y.S. (2019). Influence of fiber length and its distribution in three phase poly(propylene) composites. J. Compos. B Eng..

[12] Lienhard J., Schulenberg L. (2018). Strain rate dependent multiaxial characterization of long fiber reinforced plastic. J. Compos. B Eng..

[13] Bartus S.D., Vaidya U.K., Ulven C.A. (2006). Design and development of a long fiber thermoplastic bus seat. J. Thermoplast. Compos. Mater..

[14] Bijsterbosch H., Gaymans R.J. (1995). Polyamide 6—Long glass fiber injection moldings. J. Polym. Compos..

[15] Guan W.S., Huang H.X. (2012). Back melt flow in injection–compression molding: Effect on part thickness distribution. J. Int. Commun. Heat Mass Transf..

[16] Oh H.J., Lee D.J., Lee C.G., Jo K.Y., Lee D.H., Song Y.S., Young J.R. (2013). Warpage analysis of a micro-molded parts prepared with liquid crystalline polymer based composites. J. Compos. Part A Appl. Sci. Manuf..

[17] Li Y., Li W., Tao Y., Shao J., Deng Y., Kou H., Zhang X., Chen L. (2019). Theoretical model for the temperature dependent longitudinal tensile strength of unidirectional fiber reinforced polymer composites. J. Compos. B Eng..

[18] Zhang D., He M., Luo H., Qin S., Yu J., Guo J. (2016). Performance of long glass fiber-reinforced polypropylene composites at different injection temperature. J. Vinyl Addit. Technol..

[19] Desplentere F., Six W., Bonte H., Debrabandere E. Influence of the power law index on the fiber breakage during injection molding by numerical simulations. Proceedings of the International Conference ‘Novel Trends in Rheology V’.

[20] Rohde M., Ebel. A., Wolff-Fabris F., Altstädt V. (2011). Influence of processing parameters on the fiber length and impact properties of injection molded long glass fiber reinforced polypropylene. Int. Polym. Process.

[21] Spahr D.E., Friedrich K., Schultz J.M., Bailey R.S. (1990). Microstructure and fracture behaviour of short and long fibre-reinforced polypropylene composites. J. Mater. Sci..

[22] Kumar K.S., Bhatnagar N., Ghosh A.K. (2008). Mechanical properties of injection molded long fiber polypropylene composites, Part 2: Impact and fracture toughness. J. Polym. Compos..

[23] Hohe J., Beckmann C., Paul H. (2015). Modeling of uncertainties in long fiber reinforced thermoplastics. J. Mater. Des..

[24] Lafranche E., Krawczak P., Ciolczyk J.P., Maugey J. (2005). Injection moulding of long glass fiber reinforced polyamide 66: Processing conditions/microstructure/flexural properties relationship. J. Adv. Polym. Technol..

[25] Wieme T., Tang D., Delva L., D’hooge D.R., Cardon L. (2018). The relevance of material and processing parameters on the thermal conductivity of thermoplastic composites. J. Polym. Eng. Sci..

[26] Schweizer R.A. (2006). Glass fiber length degradation in thermoplastics processing. J. Polym. Plast. Technol. Eng..

[27] Arroyo M., Avalos F. (1989). Polypropylene/low density polyethylene blend matrices and short glass fibers based composites I. Mechanical degradation of fibers as a function of processing method. J. Polym. Compos..

[28] Franzén B., Klason C., Kubát J., Kitano T. (1989). Fibre degradation during processing of short fibre reinforced thermoplastics. J. Compos..

[29] Phelps J.H., Abd El-Rahman A.I., Kunc V., TuckerIII C.L. (2013). A model for fiber length attrition in injection-molded long-fiber composites. J. Compos. Part A Appl. Sci. Manuf..

[30] Bailey R., Rzepka B. (1991). Fibre orientation mechanisms for injection molding of long fibre composites. Int. Polym. Process.

[31] Oppelt T., Schulze J., Stein H., Platzer B. (2012). Comparison of methods for mould surface heating—Part 1: Review. Int. Polym. Sci. Technol..

[32] Wang G.L., Zhao G.Q., Guan Y.J. (2013). Thermal response of an electric heating rapid heat cycle molding mold and its effect on surface appearance and tensile strength of the molded part. J. Appl. Polym. Sci..

[33] Wang G.L., Zhao G.Q., Wang X.X. (2013). Effects of cavity surface temperature on mechanical properties of specimens with and without a weld line in rapid heat cycle molding. J. Mater. Des. Sci..

